# A Comparative Study of AI-Based International Roughness Index (IRI) Prediction Models for Jointed Plain Concrete Pavement (JPCP)

**DOI:** 10.3390/ma15165605

**Published:** 2022-08-15

**Authors:** Qiang Wang, Mengmeng Zhou, Mohanad Muayad Sabri Sabri, Jiandong Huang

**Affiliations:** 1 School of Mines, China University of Mining and Technology, Xuzhou 221116, China; 2 Peter the Great St. Petersburg Polytechnic University, 195251 St. Petersburg, Russia; 3 School of Civil Engineering, Guangzhou University, Guangzhou 510006, China

**Keywords:** JPCP, smoothness, MEPDG, machine-learning methods, hyperparameter

## Abstract

The international roughness index (IRI) can be employed to evaluate the smoothness of pavement. The previously proposed mechanical-empirical pavement design guide (MEPDG), which is used to model the IRI of joint plain concrete pavement (JPCP), has been modified in this study considering its disadvantage of low prediction accuracy. To improve the reliability of the prediction effect of the IRI for JPCP, this study compares the prediction accuracy of the IRI of JPCP by using the machine-learning methods of support vector machine (SVM), decision tree (DT), and random forest (RF), optimized by the hyperparameter of the beetle antennae search (BAS) algorithm. The results from the machine-learning process show that the BAS algorithm can effectively improve the effectiveness of hyperparameter tuning, and then improve the speed and accuracy of optimization. The RF model proved to be the one with the highest prediction accuracy among the above three models. Finally, this study analyzes the importance score of input variables to the IRI, and the results show that the IRI was proportional to all the input variables in this study, and the importance score of initial smoothness (IRI_I_) and total joint faulting cumulated per km (TFAULT) were the highest for the IRI of JPCP.

## 1. Introduction

Joint plain concrete pavement (JPCP), using shrinkage to control the cracks, is a common form of concrete pavement without any reinforcement [1]. It is widely used because of its advantages of low initial cost and maintenance cost compared with other concrete pavements [2,3,4,5,6,7,8]. The smoothness of JPCP is an important index when evaluating pavement condition, which is not only the comprehensive embodiment of pavement disease but also reflects the performance of the road [9,10,11,12,13,14]. The smoothness of pavement is an important reference index affecting driving quality, driving safety, and road service quality, so the study of pavement smoothness is of great significance in the evaluation of the performance of pavement [15,16,17,18]. Road agencies often test the parameters of the performance of pavement, and timely take appropriate maintenance of the required sections according to the detection situation, which is an important basic condition to ensure the comfort, safety, and high quality of driving [2,19,20,21]. 

Long-term Pavement Performance (LTPP) is the most comprehensive study of pavement performance data collection, covering about 900 roads in the United States, Canada, and other countries with various influencing factors, including general information, climate data, material test data, traffic data, and pavement inspection data [22]. It is of great significance for the sustainable development of JPCP to study and analyze the smoothness of JPCP through effective extraction and mining analysis of LTPP data [23]. The prediction models of pavement can be divided into two types: the prediction model of the smoothness of pavement with a single-index and the prediction model of the smoothness of pavement with a multi-index, in which the single-index pavement smoothness prediction model takes the smoothness as the dependent variable and the indexes affecting the pavement smoothness as the independent variable [24]. IRI has the advantages of strong time stability and high result validity and is the most widely used prediction index of the smoothness of pavement in the world [24,25]. 

**Figure 1:**
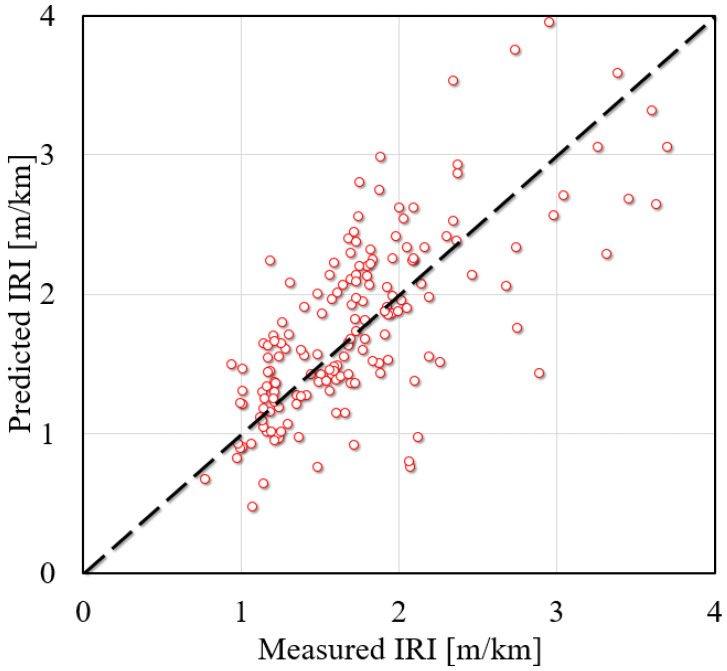
Comparison of the predicted and measured IRI results [28].

The Mechanical-Empirical Pavement Design Guide (MEPDG) is a common method for predicting the smoothness of pavement [26,27]. The prediction model of smoothness of the MEPDG is based on the LTPP database, where IRI is the evaluation index of smoothness, and initial smoothness, pavement disease type, road life, and environment are taken as dependent variables. The prediction model of the smoothness of JPCP is established according to the MEPDG as follows:

(1) $$\mathrm{IRI}={\mathrm{IRI}}_{I}+0.013\cdot \mathrm{TC}+0.007\cdot \mathrm{SPALL}+0.005\cdot \mathrm{TFAULT}+0.4S\cdot \mathrm{FT}$$

where IRI_I_ is initial smoothness measured as IRI, m/km, TC is the percentage of slabs with transverse cracking (all severities), SPALL is the percentage of joints spalled, PATCH is the pavement surface area with flexible and rigid patching (all severities) as a percentage, TFAULT is the total joint faulting cumulated per km, mm, *SF* is the site factor, and SF=AGE⋅(1+FI)⋅(1+ρ200)/1000000, *AGE* is the pavement age in years, *FI* is the freezing index, days, and ρ200 is the percentage of subgrade material passing the 0.075-mm sieve. The prediction results of the IRI of JPCP by the MEPDG are shown in Figure 1. 

It is clear from the figure that the predicted value of the IRI of JPCP by the MEPDG differs greatly from the actual value, that is, the fitting effect is poor. Therefore, it is of great practical significance to propose a more accurate and efficient model to predict the IRI of JPCP. Gulfam-E-Jannat et al. conducted a regression analysis to evaluate IRI models by comparing the predicted distress to observed distress [29]. Souliman et al. have employed local pavement distress data, material properties, traffic information, and local environmental conditions in the Arizona area to evaluate the MEPDG including the IRI prediction model [30]. The research from Saha et al. showed that the IRI was sensitive to climate changes and the differences in the quality and duration of data for close-by stations can result in variation in the predicted performance [31]. The IRI models of the MEPDG have also been calibrated using an optimization approach embedded in solver optimization in MS Excel (Microsoft, Redmond, WA, USA) with new coefficients of the IRI model [26]. It can be found from previous studies that the prediction of the IRI relying on mathematical regression can lead to inconsistent parameters obtained from different databases, and the accuracy obtained in the study was relatively low, resulting in low reliability of the above prediction models for the IRI.

With the development of machine learning, the application of machine-learning models to predict the performance of pavement has become a research hotspot for architectural engineering researchers [13,32,33,34,35,36,37,38,39,40,41,42,43,44,45,46,47]. Marcelino et al. proposed a general machine-learning method that supports different machine-learning methods and generalization performance for the development of pavement performance prediction models in pavement management systems [48]. Yan et al. established an evaluation model of the performance of asphalt pavement using SVM and optimized the model with Particle Swarm Optimization (PSO). The research results showed that the established PSO-SVM model is simple and effective, and can be used to evaluate the performance of asphalt pavement [49]. Gungor et al. established an SVM model for predicting the internal temperature, bending, and bending strain of airport instrument taxiway pavement, and the results showed that the SVM method has a higher prediction accuracy and a shorter prediction time [50]. Wang et al. proposed a prediction model of the performance of pavement based on a hybrid Grey Relation Analysis (GRA) and Support Vector Regression (SVR). The method used the genetic algorithm to screen the main factors influencing the performance of asphalt pavement, the SVR to predict the performance of asphalt pavement, and the accuracy of the model was validated with data from actual highways [51]. However, most researchers have only studied the prediction effect of a single machine-learning model on JPCP IRI, and few researchers have proposed using a hybrid machine-learning model to predict road performance and compare the prediction effect of multiple hybrid machine-learning models, so as to select the model with a better prediction effect [52,53]. In order to improve the prediction accuracy of the IRI of JPCP, this study proposes that BAS is firstly used to optimize the hyperparameters of SVM, DT and RF, and the prediction effect of the three models on the IRI of JPCP was compared, and then the prediction model with a better prediction effect on the IRI of JPCP was selected. 

## 2. Methodology

**Figure 2:**
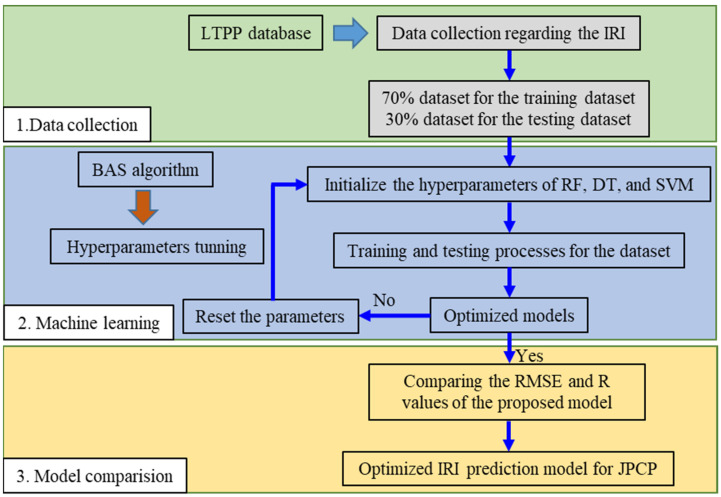
Flow chart of the research process.

Figure 2 presents the research process of this study, including the data collection, machine learning, and model comparison. The LTPP database was employed to establish the model between the input parameters and the IRI values. Seventy percent of the database was used for the training process and the remaining thirty percent was for testing. The machine-learning process was to establish the relationship between the input parameters and the IRI values, using RF, DT, and SVM, respectively. The predictive effects of these models were compared by determining the R and RMSE values of varying models. The detailed process of these research sections is described as follows.

### 2.1. Data Collection

The basis for verifying the prediction accuracy of the IRI of JPCP by machine-learning models is to establish a large and reliable database. In this study, 177 data sets were collected from the LTPP database to form a database for predicting the IRI of JPCP [54]. In the 177 data sets, the input variables were the same as the input of the IRI prediction model in the MEPDG, so that the effects of the model proposed in this study and the MEPDG model could be compared effectively. The database contained eight input variables (initial smoothness measured as IRI (IRI_I_), pavement age in years (AGE), percentage of slabs with transverse cracking (all severities) (TC), percentage of joints spalled (SPALL), pavement surface area with flexible and rigid patching (all severities) (PATCH), total joint faulting cumulated per km (TFAULT), freezing index (FI), percentage of subgrade material passing the 0.075-mm sieve ($\rho_{200}$)) and one output variable (IRI). The rationality of the data distribution was very important for the evaluation of the accuracy of the prediction model. To verify the rationality of data distribution in this study, a table of variable data distribution was made, as shown in Table 1. It can be clearly seen from the table that the data distribution of input variables was reasonable and covered a wide range, so the data distribution of the actual IRI obtained was relatively reasonable.

### 2.2. Correlation Analysis

**Figure 3:**
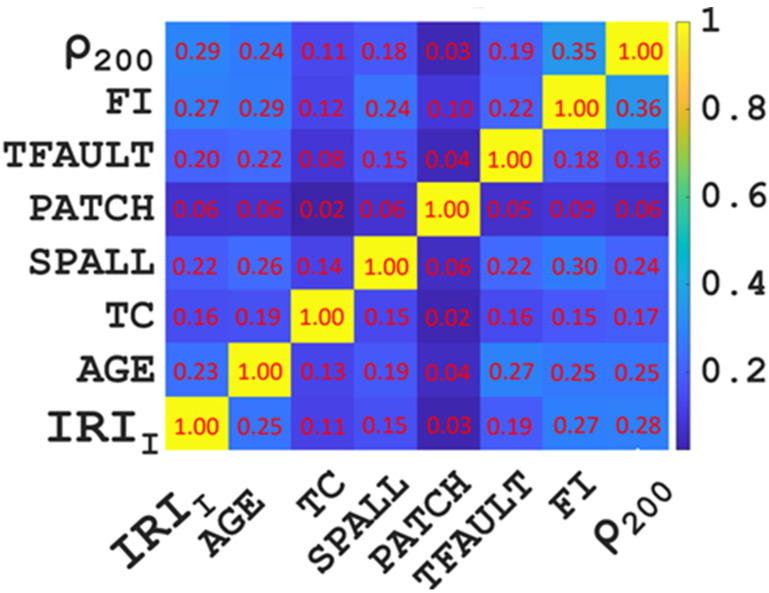
Correlation analysis results of input variables.

From previous research experience, we know that a high correlation between input variables will affect the prediction effect of the model because of the multicollinearity between variables. To avoid the above situation, a correlation analysis of input variables was carried out before the model training in this study. The correlation analysis results among the eight input variables are shown in Figure 3. We can clearly see that the same input variables were highly correlated with each other, and the correlation coefficient was 1, while the correlation coefficient between different input variables was less than 0.6. The above results prove that there was no high correlation between the 8 input variables selected in this study. Therefore, selecting them as input variables to predict the IRI of JPCP will not affect the prediction effect of the model due to the high correlation between the input variables. 

### 2.3. Modified Beetle Antennae Search (MBAS)

#### 2.3.1. Beetle Antennae Search (BAS) Algorithm

BAS is a common intelligent optimization algorithm, as well as a single-search algorithm that simulates the search mode of beetles foraging [55]. For the BAS algorithm, efficient optimization can be realized without knowing the specific form of function or gradient information. Compared with the particle-swarm optimization (PSO) algorithm, the BAS algorithm only needs one individual variable, which greatly reduces the amount of computation. Therefore, it has the characteristics of a very small amount of computation and very fast convergence. In addition, it has a global optimization ability compared with other search algorithms [56,57,58,59]. Therefore, the BAS algorithm was selected in this study to tune the hyperparameters. 

In the process of foraging, beetles decide the direction of flight according to the concentration of the food smells and feel the smell of the food through two tentacles. Because the food has a different distance from the two tentacles, the concentration of food odor received by the two antennae is different. The beetles fly in the direction of the high concentration of food odor until they finally find the food. The position of the beetle in *n*-dimensional space is defined as:

(2) $$X\left(x_{1},x_{2},\ldots ,x_{n}\right)$$

The position of the left and right antennae of the Longicorn beetle is defined as:

(3) $$\{\begin{array}{c} X_{r}=X+l\cdot \vec{d}\\ X_{l}=X-l\cdot \vec{d} \end{array}$$

where, l is the distance between the center of mass and the tentacle of the Longicorn beetle, and $\vec{d}$ is the random unit vector.

The normalized processing formula of the unit vector is as follows:

(4) $$\vec{d}=\frac{rands\left(D,1\right)}{∥rands\left(D,1\right)∥}$$

where rands(⋅) stands for random function.

The beetle position update formula is as follows:

(5) $$X_{t+1}=X_{T}+\delta_{t}\cdot \vec{d}\cdot sign\left[f\left(X_{r}\right)-f\left(X_{l}\right)\right]$$

In the above formula, *t* represents the number of iterations, *f* is the target function, $\delta_{t}$ represents the step at time *t*, and sign(⋅) is the symbolic function. In general, the step size needs to get smaller and smaller during each iteration. Set it as:

(6) $$\delta_{t+1}=\delta_{t}\cdot \eta$$

In the above formula, η represents the speed of step size setting in each iteration, which is usually 0.95.

The algorithm has the advantages of simple principle, few parameters, and less computation in dealing with low-dimensional optimization objectives.

#### 2.3.2. Support Vector Machine (SVM) Model

SVM is a kind of generalized linear classifier for the binary classification of data by supervised learning. It has been widely used in classification and regression fields. Its core idea is to find an optimal classification hyperplane. Support vector machines include linear support vector machines and nonlinear support vector machines.

For linear support vector machines, first, assume the sample set $T=\left\{\left(x_{1},y_{1}\right),\left(x_{2},y_{2}\right),\ldots \left(x_{i},y_{i}\right)\right\}$, where $x_{i}$ is the *i*th feature vector of the space, and $x_{i}\in R^{D}$, where $y_{i}$ represents the corresponding category of $x_{i}$, and $y_{i}\in \left\{-1,+1\right\}$, and assume that the samples are linearly separable in the feature space.

Assume that $g\left(x\right)=\omega^{T}+b$ is the discriminant function of the D-dimensional space, and the discriminant function has a hyperplane: H, where $\omega^{T}+b=0$. After normalization, the following equation can be obtained:

(7) $$y_{i}\left(\omega^{T}\cdot x_{i}+b\right)-1\geq 0,i=1,2,3\ldots n$$

Then, the geometric interval between the two types of samples is:

(8) $$M\arg in=\frac{2}{∥\omega ∥}$$

According to the above formula, when ∥ω∥ is minimized, the classified hyperplane is the optimal hyperplane. The optimal hyperplane problem of SVM is to find the maximum margin, that is, to find the minimum of $\frac{∥\omega^{2}∥}{2}$:

(9) $$\{\begin{array}{c} \min\frac{∥\omega {∥}^{2}}{2}\\ s.t.y_{i}\left(\omega^{T}\cdot x_{i}+b\right)-1\geq 0,i=1,2,3,\ldots n \end{array}$$

After the introduction of the Lagrange multiplier:

(10) $$L\left(\omega ,b,\alpha \right)=\frac{\omega^{T}}{2}\cdot \omega -\overset{n}{\underset{i=1}{\sum }}\alpha_{i}\left[y_{i}\left(\omega^{T}\cdot x_{i}+b\right)-1\right]$$

In the formula, *n* is the Lagrange coefficient, and $\alpha_{i}\geq 0,i=1,2,3\ldots n$ is the parameter in the above formula. Let the partial derivatives of ω,b,α be 0, and get:

(11) $$\{\begin{array}{c} \omega =\overset{n}{\underset{i=1}{\sum }}\alpha_{i}\cdot y_{i}\cdot x_{i}\\ \overset{n}{\underset{i=1}{\sum }}\alpha_{i}\cdot y_{i}=0\\ \alpha_{i}\left[y_{i}\left(\omega^{T}\cdot x_{i}+b\right)-1\right]=0 \end{array}$$

With the solution:

(12) $$\{\begin{array}{c} \max\overset{n}{\underset{i=1}{\sum }}\alpha_{i}-\frac{1}{2}\overset{n}{\underset{i=1}{\sum }}\overset{n}{\underset{j=1}{\sum }}\alpha_{i}\cdot \alpha_{j}\cdot y_{i}\cdot y_{j}\left(x_{i}^{T}\cdot x_{j}\right)\\ s.t\alpha_{i}\geq 0,i=1,2,3,\ldots n\\ \overset{n}{\underset{i=1}{\sum }}\alpha_{i}\cdot y_{i}=0 \end{array}$$

Based on the above analysis, the optimal classification discriminant function is:

(13) $$f\left(x\right)=\mathrm{sgn}(\left(\omega^{T}\cdot x\right)+b)=\mathrm{sgn}\left\{\overset{n}{\underset{i=1}{\sum }}\alpha_{i}\cdot y_{i}\cdot \left(x_{i}\cdot x\right)+b\right\}$$

Since it is difficult to find the optimal hyperplane for linear non-separable samples, the concept of a kernel function is added to the linear non-separable sample SVM. Firstly, the data need to be mapped from low-dimensional space to high-dimensional space through linear transformation, and then the classification surface is calculated. The linear transformation process formula is as follows:

(14) $$K\left(x_{i},y_{j}\right)=\langle \phi \left(x_{i}\right)\cdot \phi (x_{j})\rangle$$

The change in the optimal classification discriminant function is:

(15) $$f\left(x\right)=\mathrm{sgn}(\left(\omega^{T}\cdot \phi \left(x\right)\right)+b)=\mathrm{sgn}\left\{\overset{n}{\underset{i=1}{\sum }}\alpha_{i}y_{i}\cdot K\left(x_{i}\cdot x\right)+b\right\}$$

SVM commonly used kernel functions ($K\left(x_{i},x_{j}\right)$) mainly include the linear kernel function ($K\left(x_{i},y_{j}\right)=x_{i}\cdot x_{j}$), polynomial kernel function ($K\left(x_{i},y_{j}\right)={\left[\gamma \left(x_{i}\cdot x_{j}\right)+r\right]}^{d},\gamma >0$) Gaussian kernel function ($K\left(x_{i}\cdot x_{j}\right)=\exp(-\frac{∥x_{i}-x_{j}∥{}^{2}}{2\sigma^{2}}),\sigma >0$), and Sigmoid kernel function ($K\left(x_{i},y_{j}\right)=\tanh(\gamma \left(x_{i}\cdot x_{j}\right)+r)$), where r,γ,σ are all kernel function parameters and *d* is a positive integer.

#### 2.3.3. Decision Tree (DT) Model

DT is a graphical method that intuitively uses probability analysis. Based on the known probability of occurrence of various situations, it obtains the probability that the expectation of the net-present value is greater than or equal to zero by constructing a decision tree, evaluating the project risk, and determining the feasible decision analysis method of a prediction model. A decision tree is a tree structure in which internal nodes represent tests on attributes, branches represent test outputs, and leaf nodes represent categories of classification [60,61]. The steps of DT are as follows: first create node N. If the training set is empty, it returns the node N markers for failure. If all training records belong to the same category, with the category tag reception N, and if the candidate attribute is null, it returns N as a leaf node, and marks it as a training focus on the most common class. Then, an attribute list is defined for each candidate attribute. If the candidate attribute is continuous, the attribute is discretized. It selects the attribute D with the highest information gain rate in the attribute list of candidate attributes, and marks node N as attribute D. For the consistent value D of attribute d, a branch with condition D = d grows from node N. Let s be the training sample set D = D in the training set. Then, an attribute list is defined for each candidate attribute. If the candidate attribute is continuous, the attribute is discretized. It selects the attribute D with the highest information gain rate in the attribute list of candidate attributes, and marks node N as attribute D. For the consistent value D of attribute d, a branch with condition D = d grows from node N. Assume the training sample set D = d in the training set. If S is empty, it adds a leaf and marks it as the most common class in the training set, otherwise, it adds a new return point. 

#### 2.3.4. Random Forest (RF) Model

RF is a common classification method based on the Bagging ensemble theory, which uses a decision classification tree as a sub-classifier. RF firstly uses Bootstrap random sampling technology to extract a certain number of samples from the data set with putting-back to generate the corresponding sub-training set and test set, and then constructs an independent decision tree for each sub-training set to form a random forest. The construction steps of the random forest are as follows.

(1) Extraction of the sub-training set. By the Bootstrap method, N samples are randomly extracted from data set D, put back to form K sub-training data sets, and K decision trees are established.
(2) Construction of decision tree. The construction of subclassifies mainly uses a classification regression tree. Firstly, K features are randomly selected from M features at each node of the decision tree as the segmentation feature set of corresponding nodes. The optimal segmentation feature and segmentation node are determined according to relevant criteria. The segmentation node is divided into two nodes, and the corresponding data is also divided into two nodes. The above process is repeated until the stop condition is met.
(3) Construction of random forest. Step (2) is repeated, and stops when K decision trees are generated, and then these are combines into a random forest $\left\{t_{i},i=1,2,\ldots ,K\right\}$.
(4) K decision trees in the random forest are used to classify the test data set DT, and K prediction results ($\left\{t_{1}\left(x\right),t_{2}\left(x\right),\ldots t_{K}\left(x\right)\right\}$) are obtained.
(5) The mode in the prediction result of the decision tree is selected as the final prediction result of each sample in the prediction data set.

## 3. Results and Discussion

### 3.1. Results of the Hyperparameter Tuning 

**Figure 4:**
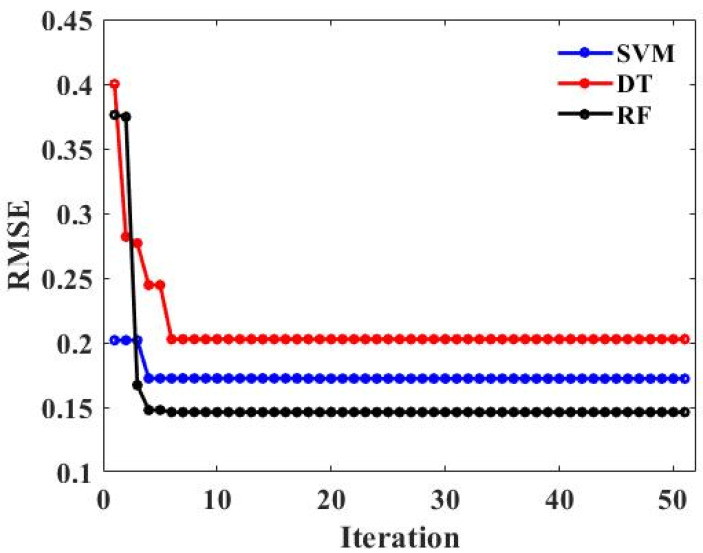
The relationship between the number of iterations of the models and RMSE values.

Tuning the hyperparameters of the model before the model training is beneficial in improving the prediction effect of the model. In this study, BAS was selected to optimize the hyperparameters of SVM, DT, and RF. The relationship between the RMSE values of the three models and the number of iterations is shown in Figure 4. During the process of hyperparameter tuning, the RMSE value gradually decreased, indicating that the predicted values obtained by the three machine-learning models were gradually approaching the measured value. The prediction process of machine-learning models tended to converge as the RMSE values tended to be stable. This also reflects that the BAS algorithm could effectively and quickly adjust the hyperparameters of the three machine-learning models. In addition, as can be seen from the below figure, the RMSE of the three models decreased sharply with the increase in the iteration number and then tended to be stable. After the RMSE tended to be stable, the RMSE value of RF in the three models was the lowest.

**Figure 5:**
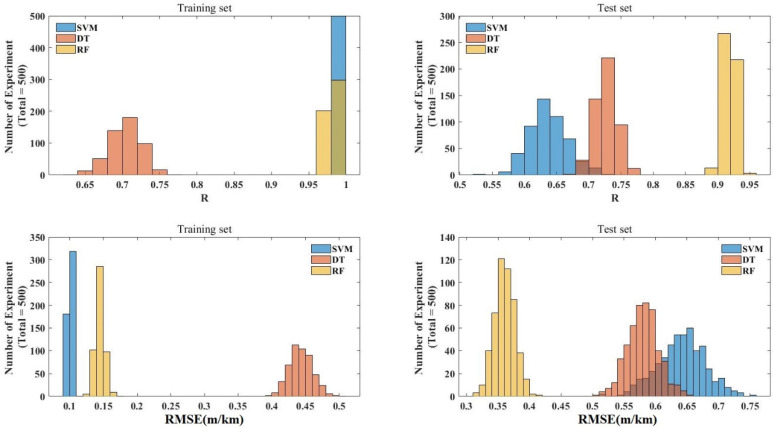
R values and RMSE values of the training set and test set of SVM, DT, and RF.

Figure 5 shows the relationship between the number of iterations of the SVM, DT, and RF models and the R values and RMSE values in the training set and the test set, respectively. The figure shows that in the training set, SVM and RF had the highest R value, while SVM had the lowest RMSE value. In the test set, RF had the highest R value and the lowest RMSE value in the three models. Although RF did not have the lowest RSME value in the training set, it had the highest R value in the training set and the highest R value, and the lowest RMSE value in the test set. In general, RF was the best machine-learning model for the prediction of the IRI of JPCP among the three machine-learning models tuned by BAS. The possible reason for this is that the RF model does not easily fall into overfitting due to the introduction of two randomness variables compared to other machine-learning models (For example, the over-fitting phenomenon of SVM appeared in this study). Another possible reason is that the RF model can process high-dimensional data without feature selection and has a strong adaptability to the IRI dataset.

**Figure 6:**
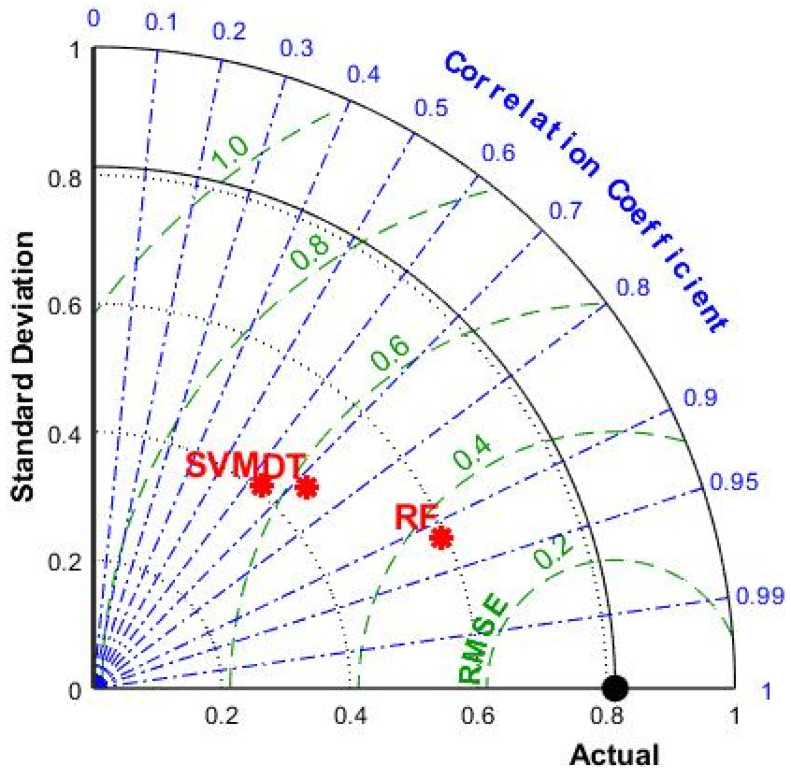
Radar plots for R values and RMSE values of SVM, DT, and RF.

Figure 6 is the radar diagram of the R value and RMSE value of the three machine-learning models after BAS hyperparameter tuning. We can clearly see that the RF model of the three models had the highest R value and the lowest RMSE value, which proves once again that the RF model was the model with the best prediction effect among the three machine-learning models in this study.

### 3.2. Comparison of the Predictive Performance 

**Figure 7:**
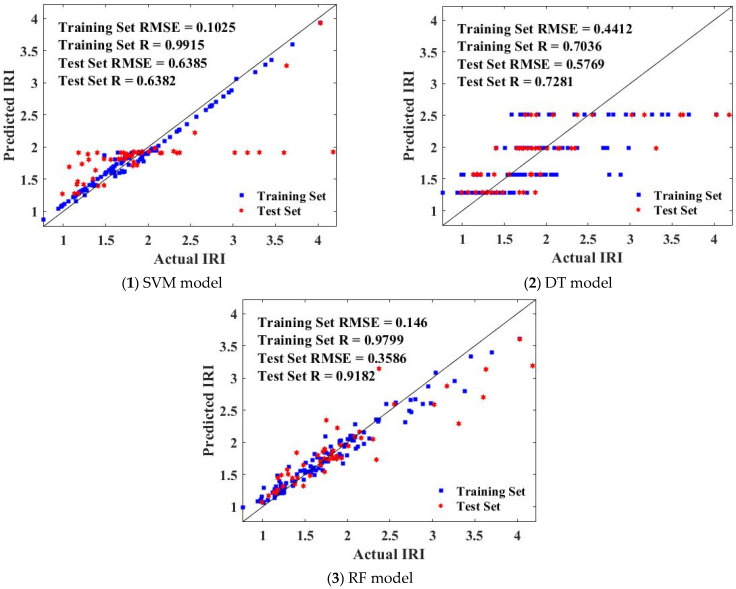
The comparison between the predicted value and the actual value of the SVM, DT, and RF.

This study evaluated the prediction effect of the above three models on the IRI of JPCP by comparing and analyzing the fitting effect of the predicted value and the actual value of SVM, DT, and RF tuned by BAS. The comparative analysis of the predicted value and actual value of the training set and test set of the three models is shown in Figure 7. It is obvious that the fitting effect of the predicted value and actual value of the SVM and RF model was good, while the deviation between the predicted value and the actual value of the IRI of JPCP was large. To further select the model with the best prediction effect between SVM and RF, it was necessary to further analyze the R value and RMSE value of their training sets and test sets. As can be seen from the below figure, the RSME values of SVM and RF in the training set were 0.1025 and 0.146 respectively, and the R values were 0.9915 and 0.9799, respectively, that is, SVM in the training set has a low RMSE value, RF has a high R value, and the R values of SVM and DT in the test set are 0.6382 and 0.9182, respectively. The RMSE values were 0.6385 and 0.3586, respectively. RF had a higher R value and a lower RMSE value in the test set. As can be seen from the below figure, the RSME values of SVM and RF in the training set were 0.1025 and 0.146, respectively, and the R values were 0.9915 and 0.9799, respectively. SVM in the training set had a low RMSE value, RF had a high R value, and the R values of SVM and DT in the test set were 0.6382 and 0.9182, respectively. The RMSE values were 0.6385 and 0.3586, respectively. RF had a higher R value and a lower RMSE value in the test set.

**Figure 8:**
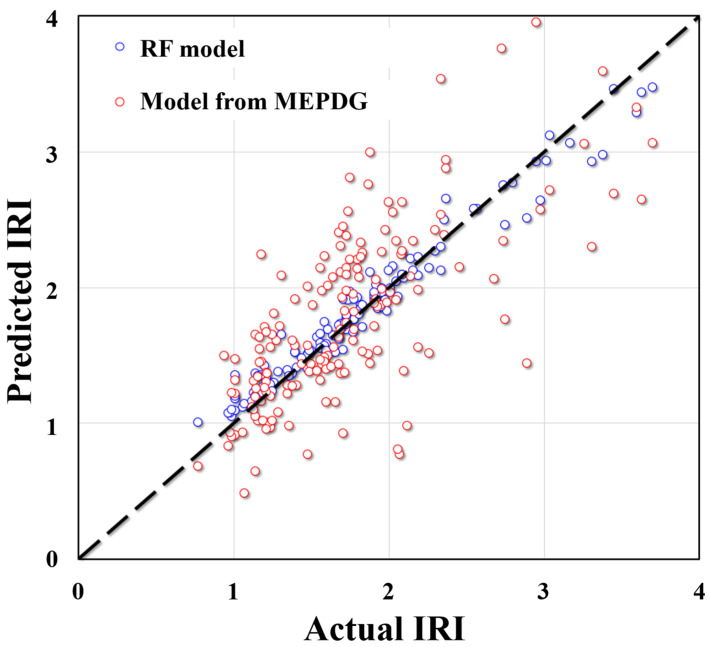
Comparison between the RF model and model from MEPDG.

Figure 8 gives the comparison between the RF model proposed in this study and the model from MEPDG. It is clear that the proposed model in this study was close to the “1:1” line compared with the MEPDG model. In addition, the MEPDG model shows great discreteness when the IRI was at a high value while the model presented in this study still had good predictive performance in a wide range of regions. It is indicated in the below figure that the hybrid RF model proposed in this study had good accuracy and model applicability. The research results formed in this study can be used in practical engineering. Engineers can use the model obtained in this study to reliably predict the IRI of future road surfaces, which can be used as a reference for road maintenance.

### 3.3. Variable Importance

**Figure 9:**
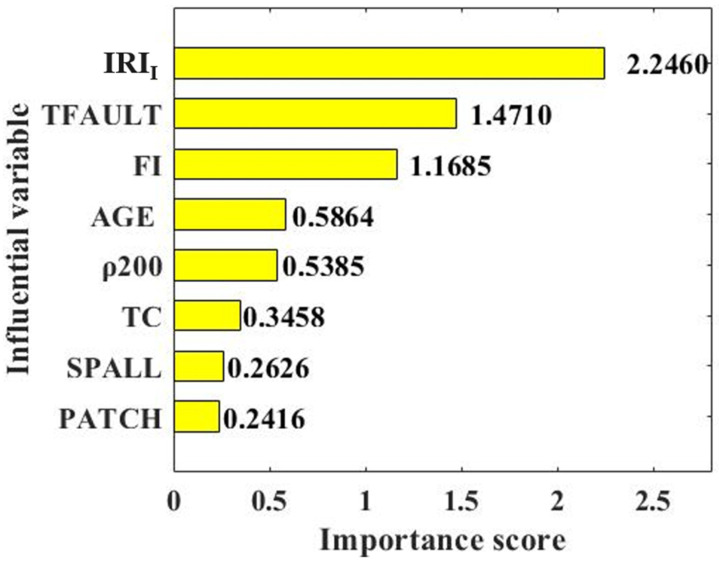
Importance score of the input variable.

To further analyze the importance of the input variables selected in this study on the IRI of JPCP, the importance scores of eight input variables were analyzed, as shown in Figure 9. The importance scores of IRI_I_, TFAULT, FI, AGE, TC, SPALL, and PATCH were 2.2460, 1.4710, 1.1685, 0.5864, 0.5385, 0.3458, 0.2626, and 0.2416, respectively. That is, the IRI of JPCP was proportional to IRI_I_, TFAULT, FI, AGE, TC, SPALL, and PATCH, and the importance of variables on the IRI of JPCP decreased according to the above order. IRI_I_ and TFAULT were the most important variables on the IRI of JPCP. It should be noted that IRI_I_ is an objective parameter that cannot be artificially designed during road maintenance. Therefore, road engineers should pay attention to TFAULT when designing JPCP, as it is of great significance in improving the IRI of JPCP. The effect of SPALL and PATCH on maintaining road surface smoothness was weak, indicated by the low important scores.

## 4. Conclusions

To address the issue of the reliability of the IRI prediction for JPCP being low, this study proposed the idea of using a hybrid machine-learning model to predict the IRI of JPCP. Firstly, this study used BAS to tune the hyperparameters of SVM, DT, and RF. Then, by comparing the prediction effect of SVM, DT, and RF optimized by BAS on the IRI of JPCP, the machine-learning model with the highest accuracy prediction was selected. Through the study of the above analysis, the following conclusions are drawn.

(1) The data of the input variables (IRI_I_, TFAULT, FI, AGE, ρ_200_, TC, SPALL, PATCH) in the database had a reasonable distribution, wide coverage, and low correlation. Therefore, using this database to predict the effects of the models, the prediction effect of the IRI of JPCP will not be affected by unreasonable data distribution and a high correlation between variables.
(2) BAS had a good hyperparameter tuning effect on SVM, DT, and RF, indicated by the fact that RMSE values could quickly converge in the process of machine learning. By comparing and analyzing the predicted value and actual value of the IRI of JPCP from the three models in the training set and test set, it was found that RF had the best prediction effect (RMSE value of 0.146 and R value of 0.9799 for the training dataset; RMSE value of 0.3586 and R value of 0.9182 for the training dataset) on the *IRI* of JPCP in general among the three machine-learning models. In addition, the RF model had no fitting phenomenon due to the introduction of two randomness variables; DT had a poor-fitting effect on the predicted value of the IRI of JPCP.
(3) IRI_I_ and TFAULT were the two most important parameters for maintaining the roughness of the road surface. Considering that IRI_I_ is an objective parameter of road surface, road engineers should pay more attention to the influence of TFAULT on road surface smoothness in the design process. The effect of SPALL and PATCH on maintaining road surface smoothness was weak, indicated by the low important scores.

Regarding future development, it is possible to consider using the present hybrid machine-learning model to predict the IRI of both JPCP and jointed reinforced concrete pavement (JRCP). In addition, the results obtained can be applied to the mixture design of cement concrete pavement by considering the IRI development of the pavement.

## Figures and Tables

**Table 1:** Variable data analysis.

Variables	Minimum	Maximum	Median	Mean	STD	Variance
IRI_I_	0.43	2.59	1.11	1.18	0.45	0.20
AGE	2.37	33.71	13.21	14.11	5.95	35.40
TC	0	65.10	0	5.23	11.97	143.17
SPALL	0	105.40	4.00	20.12	30.59	935.97
PATCH	0	559.20	0	10.58	56.51	3193.93
TFAULT	0	1902.30	95.00	264.83	381.11	145,246.20
FI	0	186.28	54.8	303.62	441.25	194,704.3
$\rho_{200}$	1	97.9	35.8	39.64	27.68	744.35
IRI	0.77	4.18	1.7	1.82	0.69	0.47

## Data Availability

Data are contained within the article.

## References

[1] Hsieh Y.-A., Yang Z., Tsai Y.-C.J. (2021). Convolutional neural network for automated classification of jointed plain concrete pavement conditions. Comput.-Aided Civ. Infrastruct. Eng..

[2] Tian K., Yang B., King D., Ceylan H., Kim S. Characterization of curling and warping influence on smoothness of jointed plain concrete pavements. Proceedings of the International Airfield and Highway Pavements Conference of the Transportation and Development Institute (T and DI) of the American Society of Civil Engineers (ASCE).

[3] Zhang S., Fan Y., Huang J., Shah S.P. (2021). Effect of nano-metakaolinite clay on the performance of cement-based materials at early curing age. Constr. Build. Mater..

[4] Xu W., Huang X., Huang J., Yang Z. (2021). Structural Analysis of Backfill Highway Subgrade on the Lower Bearing Capacity Foundation Using the Finite Element Method. Adv. Civ. Eng..

[5] Ren J., Xu Y., Zhao Z., Chen J., Cheng Y., Huang J., Yang C., Wang J. (2022). Fatigue prediction of semi-flexible composite mixture based on damage evolution. Constr. Build. Mater..

[6] Ren J., Xu Y., Huang J., Wang Y., Jia Z. (2021). Gradation optimization and strength mechanism of aggregate structure considering macroscopic and mesoscopic aggregate mechanical behaviour in porous asphalt mixture. Constr. Build. Mater..

[7] Liang X., Yu X., Chen C., Ding G., Huang J. (2022). Towards the low-energy usage of high viscosity asphalt in porous asphalt pavements: A case study of warm-mix asphalt additives. Case Stud. Constr. Mater..

[8] Huang J., Leandri P., Cuciniello G., Losa M. (2021). Mix design and laboratory characterisation of rubberised mixture used as damping layer in pavements. Int. J. Pavement Eng..

[9] Grogg M., Smith K., Williges C., Schram S. (2020). Incorporating Pavement Smoothness Benefits to Enhance the Iowa Department of Transportation’s Pavement Type Determination Process. Transp. Res. Rec..

[10] Babu A., Baumgartner S.V. Road Surface Roughness Estimation Using Polarimetric SAR Data. Proceedings of the 21st International Radar Symposium (IRS).

[11] Putra T., Husaini, Machmud M. (2020). Predicting the fatigue life of an automotive coil spring considering road surface roughness. Eng. Fail. Anal..

[12] Wang L., Yan J., Xie S., Wang C. (2020). Testing, Analysis and Comparison for Characteristics of Agricultural Field and Asphalt Road Roughness. INMATEH Agric. Eng..

[13] Chen D., Lv Z. (2022). Artificial intelligence enabled Digital Twins for training autonomous cars. Internet Things Cyber-Phys. Syst..

[14] Wang Q.-A., Zhang C., Ma Z.-G., Huang J., Ni Y.-Q., Zhang C. (2021). SHM deformation monitoring for high-speed rail track slabs and Bayesian change point detection for the measurements. Constr. Build. Mater..

[15] Robbins M., Tran N., Copeland A. (2018). Determining the Age and Smoothness of Asphalt and Concrete Pavements at the Time of First Rehabilitation using Long-Term Pavement Performance Program Data. Transp. Res. Rec..

[16] Babu A., Baumgartner S.V., Krieger G. (2022). Approaches for road surface roughness estimation using airborne polarimetric SAR. IEEE J. Sel. Top. Appl. Earth Obs. Remote Sens..

[17] Zhang Y., Zhao H.S., Lie S.T., Yao Z., Sheng Z.H., Tjhen L.S. (2018). A Simple Approach for Simulating the Road Surface Roughness Involved in Vehicle-Bridge Interaction Systems. Int. J. Struct. Stab. Dyn..

[18] Huang J., Duan T., Lei Y., Hasanipanah M. (2020). Finite Element Modeling for the Antivibration Pavement Used to Improve the Slope Stability of the Open-Pit Mine. Shock Vib..

[19] Loprencipe G., Zoccali P., Cantisani G. (2019). Effects of Vehicular Speed on the Assessment of Pavement Road Roughness. Appl. Sci..

[20] Zhao H., Jiang Q., Xie W., Li X., Yin C. (2018). Role of urban surface roughness in road-deposited sediment build-up and wash-off. J. Hydrol..

[21] Wang G., Chen S., Xia M., Zhong W., Han X., Luo B., Sabri M.M.S., Huang J. (2022). Experimental Study on Durability Degradation of Geopolymer-Stabilized Soil under Sulfate Erosion. Materials.

[22] Zavagna P., Khanal A., Souliman M. (2018). LTTP data analysis: Factors affecting pavement roughness for the state of California. J. Mater. Eng. Struct..

[23] Yildirim Y., Saygili G. (2019). Pavement smoothness of asphalt concrete overlays. Int. J. Pavement Eng..

[24] Bhattacharya B.B., Darter M.I. Calibration of Fatigue Cracking and Rutting Prediction Models in Pennsylvania Using Laboratory Test Data for Asphalt Concrete Pavement in AASHTOWare Pavement ME Design. Proceedings of the International Airfield and Highway Pavements Conference of the Transportation and Development Institute (T and DI) of the American Society of Civil Engineers (ASCE).

[25] Zhang D.-B., Li X., Zhang Y., Zhang H. (2019). Prediction Method of Asphalt Pavement Performance and Corrosion Based on Grey System Theory. Int. J. Corros..

[26] Al-Qaili A.H., Al-Solieman H. (2021). Enhancing MEPDG distress models prediction for Saudi Arabia by local calibration. Road Mater. Pavement Des..

[27] Ishikawa T., Lin T. Applicability of AASHTO MEPDG approach to flexible pavements in cold regions of Japan. Proceedings of the 16th Pan-American Conference on Soil Mechanics and Geotechnical Engineering (PCSMGE).

[28] Meegoda J.N., Gao S. (2014). Roughness Progression Model for Asphalt Pavements Using Long-Term Pavement Performance Data. J. Transp. Eng..

[29] Jannat G.E., Yuan X.-X., Shehata M. (2016). Development of regression equations for local calibration of rutting and IRI as predicted by the MEPDG models for flexible pavements using Ontario’s long-term PMS data. Int. J. Pavement Eng..

[30] Souliman M., Mamlouk M., El-Basyouny M., Zapata C.E. Calibration of the AASHTO MEPDG for flexible pavement for Arizona conditions. Proceedings of the Transportation Research Board 89th Annual Meeting.

[31] Saha J., Nassiri S., Bayat A., Soleymani H. (2014). Evaluation of the effects of Canadian climate conditions on the MEPDG predictions for flexible pavement performance. Int. J. Pavement Eng..

[32] Ashraf S. (2021). A proactive role of IoT devices in building smart cities. Internet Things Cyber-Phys. Syst..

[33] Koopialipoor M., Nikouei S.S., Marto A., Fahimifar A., Armaghani D.J., Mohamad E.T. (2019). Predicting tunnel boring machine performance through a new model based on the group method of data handling. Bull. Eng. Geol. Environ..

[34] Koopialipoor M., Ghaleini E.N., Tootoonchi H., Armaghani D.J., Haghighi M., Hedayat A. (2019). Developing a new intelligent technique to predict overbreak in tunnels using an artificial bee colony-based ANN. Environ. Earth Sci..

[35] Koopialipoor M., Fahimifar A., Ghaleini E.N., Momenzadeh M., Armaghani D.J. (2020). Development of a new hybrid ANN for solving a geotechnical problem related to tunnel boring machine performance. Eng. Comput..

[36] Hasanipanah M., Monjezi M., Shahnazar A., Armaghani D.J., Farazmand A. (2015). Feasibility of indirect determination of blast induced ground vibration based on support vector machine. Measurement.

[37] Hasanipanah M., Armaghani D.J., Amnieh H.B., Majid M.Z.A., Tahir M.M.D. (2017). Application of PSO to develop a powerful equation for prediction of flyrock due to blasting. Neural Comput. Appl..

[38] Hajihassani M., Armaghani D.J., Monjezi M., Mohamad E.T., Marto A. (2015). Blast-induced air and ground vibration prediction: A particle swarm optimization-based artificial neural network approach. Environ. Earth Sci..

[39] Armaghani D.J., Koopialipoor M., Marto A., Yagiz S. (2019). Application of several optimization techniques for estimating TBM advance rate in granitic rocks. J. Rock Mech. Geotech. Eng..

[40] Armaghani D.J., Koopialipoor M., Bahri M., Hasanipanah M., Tahir M.M. (2020). A SVR-GWO technique to minimize flyrock distance resulting from blasting. Bull. Eng. Geol. Environ..

[41] Armaghani D.J., Hatzigeorgiou G.D., Karamani C., Skentou A., Zoumpoulaki I., Asteris P.G. (2019). Soft computing-based techniques for concrete beams shear strength. Procedia Struct. Integr..

[42] Armaghani D.J., Hasanipanah M., Mohamad E.T. (2016). A combination of the ICA-ANN model to predict air-overpressure resulting from blasting. Eng. Comput..

[43] Armaghani D.J., Asteris P.G. (2021). A comparative study of ANN and ANFIS models for the prediction of cement-based mortar materials compressive strength. Neural Comput. Appl..

[44] Xu W., Huang X., Yang Z., Zhou M., Huang J. (2022). Developing Hybrid Machine Learning Models to Determine the Dynamic Modulus (E*) of Asphalt Mixtures Using Parameters in Witczak 1-40D Model: A Comparative Study. Materials.

[45] Wu X., Zhu F., Zhou M., Sabri M.M.S., Huang J. (2022). Intelligent Design of Construction Materials: A Comparative Study of AI Approaches for Predicting the Strength of Concrete with Blast Furnace Slag. Materials.

[46] Wang Q.-A., Zhang J., Huang J. (2021). Simulation of the Compressive Strength of Cemented Tailing Backfill through the Use of Firefly Algorithm and Random Forest Model. Shock Vib..

[47] Ma H., Liu J., Zhang J., Huang J. (2021). Estimating the Compressive Strength of Cement-Based Materials with Mining Waste Using Support Vector Machine, Decision Tree, and Random Forest Models. Adv. Civ. Eng..

[48] Marcelino P., de Lurdes Antunes M., Fortunato E., Gomes M.C. (2021). Machine learning approach for pavement performance prediction. Int. J. Pavement Eng..

[49] Yan K.-Z., Zhang Z. Research in Analysis of Asphalt Pavement Performance Evaluation Based on PSO-SVM. Proceedings of the International Conference on Civil Engineering and Transportation (ICCET 2011).

[50] Gungor O.E., Al-Qadi I.L. (2018). Developing Machine-Learning Models to Predict Airfield Pavement Responses. Transp. Res. Rec..

[51] Wang X., Zhao J., Li Q., Fang N., Wang P., Ding L., Li S. (2020). A Hybrid Model for Prediction in Asphalt Pavement Performance Based on Support Vector Machine and Grey Relation Analysis. J. Adv. Transp..

[52] Zhu F., Wu X., Zhou M., Sabri M.M.S., Huang J. (2022). Intelligent Design of Building Materials: Development of an AI-Based Method for Cement-Slag Concrete Design. Materials.

[53] Huang J., Zhou M., Zhang J., Ren J., Vatin N.I., Sabri M.M.S. (2022). Development of a New Stacking Model to Evaluate the Strength Parameters of Concrete Samples in Laboratory. Iran. J. Sci. Technol. Trans. Civ. Eng..

[54] Hall K.T., Correa C.E., Simpson A.L. (2002). LTPP Data Analysis: Effectiveness of Maintenance and Rehabilitation Options.

[55] Huang J., Duan T., Zhang Y., Liu J., Zhang J., Lei Y. (2020). Predicting the Permeability of Pervious Concrete Based on the Beetle Antennae Search Algorithm and Random Forest Model. Adv. Civ. Eng..

[56] Huang J., Zhou M., Yuan H., Sabri M.M.S., Li X. (2022). Prediction of the Compressive Strength for Cement-Based Materials with Metakaolin Based on the Hybrid Machine Learning Method. Materials.

[57] Huang J., Zhou M., Yuan H., Sabri M.M.S., Li X. (2022). Towards Sustainable Construction Materials: A Comparative Study of Prediction Models for Green Concrete with Metakaolin. Buildings.

[58] Huang J., Zhou M., Sabri M.M.S., Yuan H. (2022). A Novel Neural Computing Model Applied to Estimate the Dynamic Modulus (DM) of Asphalt Mixtures by the Improved Beetle Antennae Search. Sustainability.

[59] Huang J., Zhang J., Gao Y. (2021). Intelligently Predict the Rock Joint Shear Strength Using the Support Vector Regression and Firefly Algorithm. Lithosphere.

[60] Huang J., Sabri M.M.S., Ulrikh D.V., Ahmad M., Alsaffar K.A.M. (2022). Predicting the Compressive Strength of the Cement-Fly Ash–Slag Ternary Concrete Using the Firefly Algorithm (FA) and Random Forest (RF) Hybrid Machine-Learning Method. Materials.

[61] Lv Z., Zhang S., Xiu W. (2020). Solving the Security Problem of Intelligent Transportation System with Deep Learning. IEEE Trans. Intell. Transp. Syst..

